# A Cell System-Assisted Strategy for Evaluating the Natural Antioxidant-Induced Double-Stranded DNA Break (DSB) Style

**DOI:** 10.3390/genes14020420

**Published:** 2023-02-06

**Authors:** Yuduki Someya, Sakine Kobayashi, Kazuya Toriumi, Shigeki Takeda, Noritaka Adachi, Aya Kurosawa

**Affiliations:** 1 Faculty of Science and Technology, Gunma University, Kiryu 376-8515, Japan; 2 Graduate School of Nanobioscience, Yokohama City University, Yokohama 236-0027, Japan; 3 Gunma University Center for Food and Science and Wellness, Gunma University, Kiryu 376-8515, Japan

**Keywords:** non-homologous end-joining, DNA double-strand break, DNA single-strand break, natural antioxidant, antitumor effect

## Abstract

Natural antioxidants derived from plants exert various physiological effects, including antitumor effects. However, the molecular mechanisms of each natural antioxidant have not yet been fully elucidated. Identifying the targets of natural antioxidants with antitumor properties in vitro is costly and time-consuming, and the results thus obtained may not reliably reflect in vivo conditions. Therefore, to enhance understanding regarding the antitumor effects of natural antioxidants, we focused on DNA, one of the targets of anticancer drugs, and evaluated whether antioxidants, e.g., sulforaphane, resveratrol, quercetin, kaempferol, and genistein, which exert antitumor effects, induce DNA damage using gene-knockout cell lines derived from human Nalm-6 and HeLa cells pretreated with the DNA-dependent protein kinase inhibitor NU7026. Our results suggested that sulforaphane induces single-strand breaks or DNA strand crosslinks and that quercetin induces double-strand breaks. In contrast, resveratrol showed the ability to exert cytotoxic effects other than DNA damage. Our results also suggested that kaempferol and genistein induce DNA damage via unknown mechanisms. Taken together, the use of this evaluation system facilitates the analysis of the cytotoxic mechanisms of natural antioxidants.

## 1. Introduction

Natural antioxidants in vegetables and fruits exhibit various bioactivities [1,2,3,4]. For example, sulforaphane, an antioxidant found in many cruciferous vegetables, is known to be beneficial to human health and to exert antitumor effects [5]. The combination of sulforaphane and anticancer drugs reduces side effects in both animal and 3D in vitro models [6]. Moreover, sulforaphane analogs inhibit progression of pancreatic cancer without side effects [7], suggesting that natural antioxidants and their derivatives can be applied successfully as pharmaceuticals for effective cancer therapy. However, the mechanisms of the antitumor effect of each of these natural antioxidants are not fully understood. For example, the cytotoxicity of sulforaphane inhibits poly (ADP-ribose) polymerase 1, which is involved in DNA single-strand break (SSB) repair, resulting in genomic instability in HeLa S3 cells [8]. Sulforaphane also enhances radiosensitivity by reducing the protein levels of Ku70, Ku80, and XRCC4, which are involved in non-homologous end-joining (NHEJ), a DNA double-strand break (DSB) repair pathway in human pancreatic cancer cell lines, such as Panc-1 [9]. Although sulforaphane causes genomic instability via its effects on DNA repair factors in both cases, the impact of SSBs and DSBs on cells and their repair mechanisms differs according to the type of damage [10,11]. As DNA is one of the main targets in cancer chemotherapy, such natural antioxidant-induced genomic instability mechanisms are assumed to be responsible for their cytotoxic effects on cancer cells [8]. However, the extent of DNA damage is highly variable. Therefore, to clarify the antitumor effects of natural antioxidants, it is essential to determine whether antioxidants target DNA, the type of DNA damage they induce, and whether they inhibit the repair of the aforementioned DNA damage.

Identifying the targets of a chemical using in vitro analysis is complicated, costly, and time-consuming and does not necessarily reflect its physiological effects in cells in vivo. Comet assays, pulse-field gel electrophoresis, and single-molecule Förster resonance energy transfer are often used to elucidate the DNA strand-break-inducing effects of any chemical [12,13,14]. However, it is difficult to establish the required experimental conditions. Further, these methods require specialized equipment. Therefore, a possible solution to this problem is to develop simple cell-based evaluation methods.

Among DNA damage types, such as base damage, DNA crosslinking, and strand breaks, DSBs, in which both strands of DNA are broken, are the most severe [15,16]. Further, DSBs are not only caused by ionizing radiation (IR) or DNA topoisomerase II (Top2) inhibitors but also by replication fork collisions with accumulated SSBs or during the repair process of crosslinked DNA [17,18,19]. IR- and Top2 inhibitor-induced DSBs have two DSB ends (two-ended), whereas replication-associated DSBs have only one DSB end (one-ended). The cell-killing effects of these breaks depend on whether the resulting DSB is a two- or a one-ended DSB. DSBs are directly joined to broken DNA ends by either NHEJ or alternative end-joining (a-EJ) or repaired by homologous recombination (HR) using the sister chromatid as a template [15,16,20]. When two-ended DSBs occur, repair by NHEJ, HR, or a-EJ is important for cell survival. However, in the case of one-ended DSBs, as these replication-associated DSBs do not have an appropriate partner for ligation, repair by NHEJ or a-EJ leads to cell death [17,18]. Therefore, evaluating natural antioxidants to determine whether they can induce two- or one-ended DSBs is the first step toward elucidating the mechanisms by which they induce genomic instability.

The use of cells deficient of or with inhibited DSB repair is effective for cell-based evaluations of the ability of natural antioxidants to induce DNA damage. Previously, we have generated *LIG4^−/−^* cells (NHEJ-deficient cells) and *RAD54^−/−^* cells (HR-deficient cells) originating from the human pre-B cell line, Nalm-6 [18]. In this study, we classified the sensitivity of these cells to known DNA-damage-inducing agents and on this basis evaluated the ability of some natural antioxidants to induce DNA damage using Nalm-6 and its derivative *LIG4^−/−^* and *RAD54^−/−^* cells. We also examine whether similar results are obtained using another cell line (HeLa cells) pretreated with a DNA repair inhibitor. Thus, we show that the genetic analysis performed in this study can be useful in assessing the DNA-damage-inducing ability of natural antioxidants.

## 2. Materials and Methods

### 2.1. Cell Culture

The human pre-B cell line, Nalm-6, and its derivative were cultured in a 5% CO_2_ incubator at 37 °C in minimum essential medium (MEM; Nissui Seiyaku, Tokyo, Japan) supplemented with 10% heat-inactivated fetal bovine serum (GE Healthcare, Marlborough, MA, USA), 2 mM MEM non-essential amino acids (Wako Pure Chemical, Osaka, Japan), 1 mM sodium pyruvate (Wako Pure Chemical), 50 μM 2-mercaptoethanol (Wako Pure Chemical), and 0.15 mM vitamin B12 (Sigma-Aldrich, St. Louis, MO, USA) [21]. The human cervical cancer cell line, HeLa, was obtained from the RIKEN Bioresource Research Center and cultured in a 5%-CO_2_ incubator at 37 °C in Dulbecco’s modified Eagle’s medium (Sigma-Aldrich) supplemented with 10% heat-inactivated fetal bovine serum (GE Healthcare) and 100 U/mL of penicillin/streptomycin (Wako Pure Chemical).

### 2.2. Growth Inhibition Assay

The growth inhibition assay was performed as described previously [21]. Briefly, 2 × 10^4^ cells were seeded into 24-well plates and cultured for 96 or 192 h in a growth medium containing various concentrations of bleomycin (Wako Pure Chemical), etoposide (BioVision, Mountain View, CA, USA), cisplatin (Sigma-Aldrich), camptothecin (Sigma-Aldrich), sulforaphane (Cayman Chemical Company, Ann Arbor, MI, USA), resveratrol (Wako Pure Chemical), quercetin (Sigma-Aldrich), kaempferol (Sigma-Aldrich), or genistein (Wako Pure Chemical). Subsequently, cell growth was measured using the CellTiter-Glo Luminescent Cell Viability Assay Kit (Promega, Fitchburg, WI, USA).

The colony formation assay was performed as described previously [21]. Briefly, 1 × 10^2^–10^5^ cells were seeded into 60 cm dishes containing 5 mL of agarose medium with various concentrations of methyl methanesulfonate (MMS; Sigma-Aldrich). After incubation for 2 weeks at 37 °C, visible colonies were counted, and the survival percentage was calculated by comparing the number of surviving colonies with that corresponding to the untreated controls.

### 2.3. Sensitivity Assay with NHEJ Inhibition

A sensitivity assay with NHEJ inhibition and antioxidant and cytotoxicity assays were performed based on the colony formation assay. Briefly, 1 × 10^2^ HeLa cells were seeded into 6-well plates and cultured for 24 h. Following incubation, for the sensitivity assay with NHEJ inhibition, the cells were treated with 10 μM NU7026 (Merck, Whitehouse Station, NJ, USA) for 1 h, and then 50 nM etoposide, 13 nM camptothecin, 9 μM sulforaphane, 20 μM resveratrol, 100 μM quercetin, 5 μM kaempferol (Wako Pure Chemical), or 10 μM genistein was added to the medium. After culturing for 13–14 days, the resulting colonies were fixed with 10% formaldehyde in saline, stained with 0.1% crystal violet (Wako Pure Chemical), and counted. The survival rate of the cells was calculated as the number of drug-resistant colonies (*D*) divided by the number of plated cells (*P*) and then multiplied by the plating efficiency (*PE*), as shown in the formula below.

$$Survival\;rate\;\left(\%\right)=\frac{D}{P}\times PE\times 100$$

### 2.4. Immunofluorescence Staining

Immunofluorescence staining was performed as described previously [18,22]. Briefly, HeLa cells cultured on glass coverslips were treated with 100 μM quercetin, 20 μM genistein, or 100 μM etoposide for 1 or 24 h, fixed with 4% paraformaldehyde for 15 min at 25 °C, washed three times with phosphate-buffered saline (PBS), and permeabilized with PBS/0.1% Triton X-100 for 1 h at 25 °C. The cells were then blocked for 1 h at 25 °C with PBS/0.1% Triton X-100/10% fetal bovine serum and incubated with anti-γH2AX antibody (JBW301; Merck, Darmstadt, Germany) overnight at 4 °C. Thereafter, the cells were washed with PBS and incubated with goat anti-mouse Alexa Fluor 488 antibody (Thermo Fisher Scientific, Waltham, MA, USA) for 1 h at 25 °C. Next, the cells were washed with PBS, stained with DAPI (Thermo Fisher Scientific) for 10 min, embedded in the VECTASHIELD Antifade Mounting Medium (Vector Laboratories, Burlingame, CA, USA), and examined using an All-in-One Fluorescence Microscope BZ-9000 (Keyence, Osaka, Japan).

### 2.5. Statistical Analyses

Statistical analyses were performed using Microsoft Excel. Student’s *t*-test was also performed to analyze data related to sensitivity assay owing to NHEJ inhibition. *p* < 0.05 was considered significant.

## 3. Results and Discussion

To clarify the relationship between DNA damage and the type of resulting DSBs, we first examined the sensitivity of *LIG4^−/−^* cells and *RAD54^−/−^* cells generated from the Nalm-6 cell line to known DNA-damage-inducing agents. The evaluation was performed via growth inhibition assays, based on the treatment of the cells with the selected drugs for 96 h. Drug sensitivity, in which no difference was observed between wild-type and knockout cells, was analyzed via colony formation assay. A one-ended DSB inducer, such as camptothecin, often affects colony size. When the sizes of the colonies were too small for colonies to be counted in the colony formation assay, drug sensitivity was re-analyzed via growth inhibition assays (drug treatment for 192 h).

**Figure 1:**
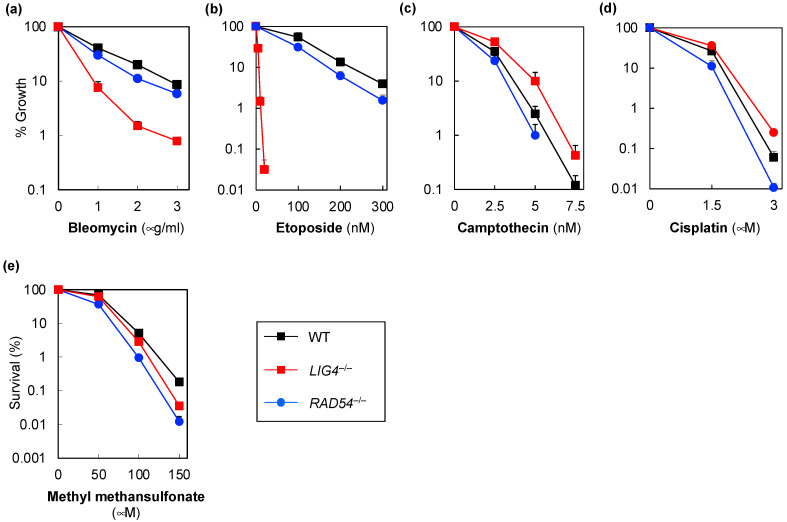
Sensitivity of human *LIG4^−/−^* (non-homologous end-joining-deficient cells), *RAD54^−/−^* (homologous recombination-deficient cells), and wild-type Nalm-6 cells to DNA damage agents. Sensitivities of human *LIG4^−/−^*, *RAD54^−/−^*, and wild-type Nalm-6 cells to bleomycin (**a**), etoposide (**b**), camptothecin (**c**), cisplatin (**d**), and methyl methanesulfonate (**e**) determined using a growth inhibition assay (**a**–**d**) or colony formation assay (**e**). Data represent the mean ± standard deviation of three independent experiments. Where absent, error bars fall within the symbols.

We previously reported that both *LIG4^−/−^* and *RAD54^−/−^* cells are more sensitive to X-rays, the radiation mimetic neocarzinostatin, and the Top2 inhibitor etoposide, compared with wild-type cells [18]. Consistent with this previous finding, both *LIG4^−/−^* and *RAD54^−/−^* cells were more sensitive to the radiomimetic agents bleomycin and etoposide (both of which are DSB inducers) than wild-type cells (Figure 1a,b). In particular, *LIG4^−/−^* cells exhibited hypersensitivity to etoposide (Figure 1b). Thus, any antioxidant toward which *LIG4^−/−^* cells show hypersensitivity may potentially be a Top2 inhibitor. However, as previously reported, *LIG4^−/−^* cells were more resistant to the DNA Topoisomerase 1 (Top1) inhibitor camptothecin (an SSB inducer) than wild-type cells, while *RAD54^−/−^* cells showed greater sensitivity in this regard to wild-type cells (Figure 1c) [18]. Further, the DNA strand crosslinker cisplatin causes 1,2-d(GpG), 1,2-d(ApG), and 1,3-d(GpNpG) intrastrand crosslinks and 1,2-d(GpC) interstrand crosslinks (ICLs) in genomic DNA [23], and nucleotide excision repair (NER), the Fanconi anemia pathways, and HR are involved in their repair [24]. Thus, *RAD54^−/−^* cells are expected to be more sensitive to cisplatin than wild-type cells. As the repair of ICLs by NHEJ can result in toxic repair [25], *LIG4^−/−^* cells are expected to be more resistant to cisplatin than wild-type cells. As expected, similar to their sensitivity to camptothecin, *LIG4^−/−^* cells were found to be more resistant to cisplatin than wild-type cells, while *RAD54^−/−^* cells were more sensitive to cisplatin than wild-type cells (Figure 1d). The alkylating agent, MMS, alkylates guanine and adenine to 7-methylguanine and 3-methyladenine, respectively [26]. Further, the alkylated base is repaired via base excision repair (BER), and in our previous study, we reported that accumulated base damage not repaired by BER is converted to DSBs in BER-proficient Nalm-6 cells and that at least some DSBs are repaired by NHEJ [18,21]. One-ended DSBs also occur in MMS-treated human A549 cells [27]. Consistent with these previous reports, *LIG4^−/−^* and *RAD54^−/−^* cells were more sensitive to the alkylating agent, MMS, than wild-type cells (Figure 1e), even though comparing the sensitivity of *LIG4^−/−^* and *RAD54^−/−^* cells to MMS revealed greater sensitivity for *RAD4^−/−^* cells than *LIG4^−/−^* cells in this regard. Thus, our results suggest that two-ended DSB has more impact on cell survival than one-ended DSB derived from SSBs that occur transiently during BER repair in Nalm-6 cells. Our results also revealed that alkylating agents constitute a class of candidate species that can be used as two-ended DSB-inducing agents in the analysis of any natural antioxidants using Nalm-6 and its derivative knockout cells. These results indicate that IR, radiomimetic agents, Top2 inhibitors, and alkylating agents induce two-ended DSBs, whereas Top1 inhibitors and DNA intrastrand and interstrand crosslinking agents induce one-ended DSBs. In Figure 1, *RAD54^−/−^* cells show higher sensitivity to all drugs than wild-type cells, suggesting that the change in the sensitivity of *LIG4^−/−^* cells is a key determinant of the DSB type. Therefore, it could be suggested that the sensitivity of *LIG4^−/−^* cells to any natural antioxidant is important for assessing whether any given natural antioxidant causes either two- or one-ended DSBs. Based on these results and our previous reports, DNA damage and the types of DSBs induced are summarized in Table 1 below.

**Figure 2:**
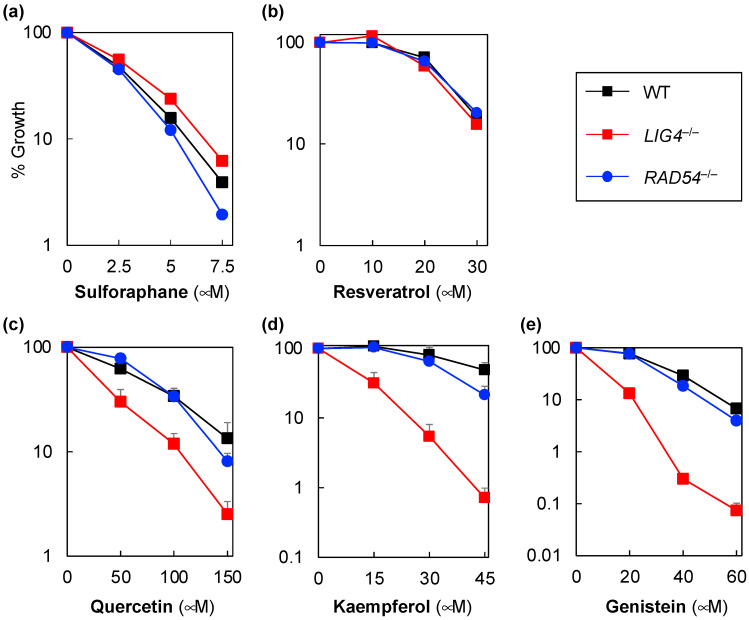
Sensitivity of human *LIG4^−/−^* (non-homologous end-joining-deficient cells), *RAD54^−/−^* (homologous recombination-deficient cells), and wild-type Nalm-6 cells to natural antioxidants. Sensitivities of human, *LIG4^−/−^*, *RAD54^−/−^*, and wild-type Nalm-6 cells to sulforaphane (**a**), resveratrol (**b**), quercetin (**c**), kaempferol (**d**), and genistein (**e**) determined using a growth inhibition assay. The cells were treated with the drug for either 96 h (**a**,**c**–**e**) or 192 h (**b**). Data are presented as the mean ± standard deviation of three independent experiments. Where absent, error bars fall within the symbols.

Next, we analyzed the sensitivity of *LIG4^−/−^* and *RAD54^−/−^* cells to five antioxidants with reported antitumor properties (sulforaphane, resveratrol, quercetin, kaempferol, and genistein) to determine whether these antioxidants possess DNA-damage-inducing ability. Sulforaphane may be involved in SSB induction or DNA crosslinking, as *LIG4^−/−^* cells were more resistant to sulforaphane than wild-type cells (Figure 2a). Further, sulforaphane can affect the factors involved in DNA SSB repair and NHEJ [8,9]. Our evaluation in this regard suggested that it may be involved in SSB induction and the inhibition of its repair, indicating that it has DNA crosslinking properties.

Since resveratrol is involved in the inhibition of DNA repair pathways that result in SSBs during repair processes, such as BER, NER, and DSB repair [30], we expected that *LIG4^−/−^* and *RAD54^−/−^* cells would be more sensitive to resveratrol than wild-type cells. However, even after 192 h of treatment with resveratrol, *LIG4^−/−^* and *RAD54^−/−^* cells were as sensitive as wild-type cells (Figure 2b). Consequently, at the treatment concentrations used in this study, our results suggested that the previously reported inhibition of DNA repair may not be the primary cause of the cytotoxicity of resveratrol.

Quercetin, kaempferol, and genistein, all of which have been suggested to be Top2 inhibitors in vitro, are structurally similar flavonoids [31,32,33]. Consistent with these reports, *LIG4^−/−^* cells showed higher sensitivity to kaempferol, quercetin, and genistein than wild-type cells (Figure 2c–e). Further, comparing the sensitivity of *LIG4^−/−^* and *RAD54^−/−^* cells to kaempferol, quercetin, and genistein revealed that *LIG4^−/−^* cells were more sensitive to these antioxidants than *RAD54^−/−^* cells. In comparison with the results shown in Figure 1, it is possible that these antioxidants induce two-ended DSBs as IR, radiomimetic agents, or Top2 inhibitors rather than as alkylating agents.

**Figure 3:**
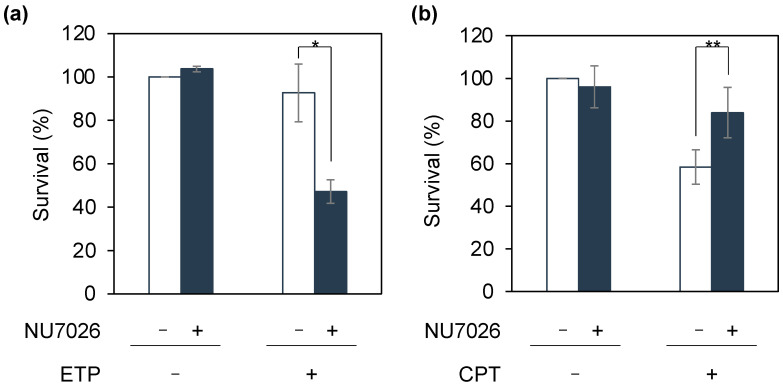
Sensitivity assay with the DNA-dependent protein kinase inhibitor, NU7026. HeLa cells were pretreated with NU7026 for 1 h prior to treatment with 50 nM etoposide (ETP, (**a**)) or 13 nM camptothecin (CPT, (**b**)). The cells were cultured for 13–14 days, and the resulting colonies were stained with crystal violet and counted. Data are presented as the mean ± standard deviation of three independent experiments. Data represent the mean ± standard deviation of at least three independent experiments (Student’s *t*-test; * *p* = 0.0054, ** *p* = 0.007). Where absent, error bars fall within the symbols.

Subsequently, we investigated whether similar results could be obtained using a cell line different from the pre-B cell line, Nalm-6. We thus performed a similar analysis involving HeLa cells using an inhibitor of NHEJ repair. Although several specific inhibitors for each NHEJ factor have been reported [34], specific inhibitors for the DNA-dependent protein kinase catalytic subunit (DNA-PKcs), one of the core factors of NHEJ, have been developed most frequently, and a number of inhibitors are commercially available [35]. Therefore, we examined whether the treatment of cells with NU7026, a DNA-PKcs inhibitor, could change the sensitivity of the cells to DNA-damage-inducing agents. We treated HeLa cells with etoposide or camptothecin with or without NU7026 pretreatment. The viability of etoposide-treated HeLa cells was significantly decreased with NU7026 pretreatment, whereas that of camptothecin-treated HeLa cells increased with NU7026 pretreatment compared with that of the control (Figure 3). In both experiments, survival rate changes with NU7026 treatment were statistically significant (*p* = 0.0054 and *p* = 0.007, respectively). Therefore, the type of DNA strand breaks was distinguished based on alterations in the sensitivity of HeLa cells to chemicals following pretreatment with NU7026.

**Figure 4:**
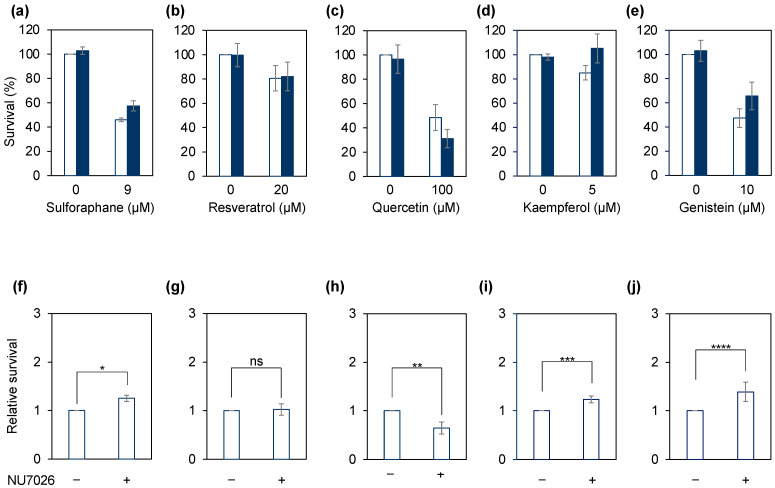
Sensitivity of HeLa cells to natural antioxidants with NU7026 pretreatment. HeLa cells were treated with or without NU7026 for 1 h and treated with sulforaphane (**a**,**f**), resveratrol (**b**,**g**), quercetin (**c**,**h**), kaempferol (**d**,**i**), or genistein (**e**,**j**). The resulting colonies were stained using crystal violet and counted. (**a**–**e**) The survival rate of HeLa cells treated with both NU7026 and natural antioxidants; the viability of the untreated cells is 100%. (**f**–**j**) Relative survival rate of HeLa cells treated with both NU7026 and natural antioxidants; the viability of the HeLa cells treated with natural antioxidants only is 1. Data represent the mean ± standard deviation of at least three independent experiments (Student’s *t*-test; * *p* = 0.0009, ** *p* = 0.0003, *** *p* = 0.005, **** *p* = 0.03, ns = no significant difference). Where absent, error bars fall within the symbols.

**Figure 5:**
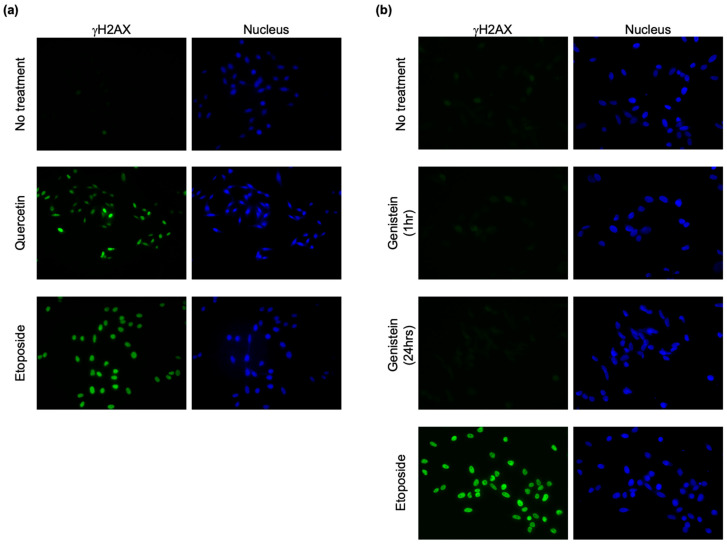
Immunofluorescence analysis of γH2AX. HeLa cells were treated with 100 μM quercetin (**a**) or 20 μM genistein (**b**) and then subjected to immunofluorescence analysis. Etoposide was used as control. Nuclei were stained with DAPI. Experiments were independently performed three times.

Finally, we examined whether the DNA-damage-inducing ability of natural antioxidants could be evaluated in HeLa cells as well as in Nalm-6 cell-derived gene-knockout cells. In this regard, we assessed the sensitivity of HeLa cells to natural antioxidants with or without NU7026 pretreatment (Figure 4a–e) and determined the relative survival of the HeLa cells treated with both the natural antioxidants and NU7026, where the survival rate of HeLa cells treated with natural antioxidants only was equal to one (Figure 4f–j). Consistent with the analysis results of the analysis of Nalm-6-derived gene-knockout cells, the survival rate of HeLa cells treated with sulforaphane significantly increased with NU7026 pretreatment (Figure 4a,f), whereas the survival rate of HeLa cells treated with resveratrol was not altered by NU7026 pretreatment (Figure 4b,g). Further, NU7026 pretreatment significantly reduced the survival rate of HeLa cells treated with quercetin (Figure 4c,h). However, the survival rate of HeLa cells treated with kaempferol or genistein increased with NU7026 pretreatment (Figure 4d,e,i,j). As γH2AX (histone H2AX, in which the 139th serine is phosphorylated) is an indicator of DSBs [36], we examined whether γH2AX is observed with quercetin or genistein treatment. As shown in Figure 5, γH2AX was observed when HeLa cells were treated with quercetin but was absent when they were treated with genistein for 24 h. The type of DNA damage induced by kaempferol and genistein and their mechanism of action remains unknown at this time. Representative Top2 inhibitors, such as etoposide, induce DSBs by stabilizing the cleavage complex. Given that γH2AX was detected in quercetin-treated HeLa cells, it was necessary to consider the possibility that DSB induction by quercetin may be due to factors other than Top2 inhibition as well as Top2. Therefore, one possibility is genistein and kaempferol are Top2 inhibitors that do not induce DSBs, similar to the Top2 inhibitor ICRF-193 [37]. Quercetin, kaempferol, and genistein have similar chemical structures; however, kaempferol and genistein exert similar cytotoxic effects, whereas quercetin exerts a different cytotoxic effect.

We observed that the genistein sensitivity of wild-type Nalm-6 cells is increased by NU7026 treatment, which differs from the results of HeLa cells with NU7026, in our preliminary experiment (Supplementary Figure S1). Therefore, it is necessary to analyze using different cell lines. However, there are still areas for improvement in this system. For example, NU7026 was used as the DNA-PK inhibitor in this study; however, a more specific inhibitor, such as M3814, may be used to obtain clearer results [35].

The results of previously reported studies as well as those of the current study will facilitate the analysis of the mechanisms underlying the antitumor effects of natural antioxidants. However, as the induction of DNA strand breaks is not the only cytotoxic mechanism of anticancer drugs, it is necessary to develop another evaluation system for natural antioxidants, such as resveratrol, which exert other antitumor effects that are not caused by DNA damage, to elucidate the mechanism of their antitumor effects. Therefore, evaluation using two different systems may be useful for elucidating the DNA-damage-inducing effects of antioxidants.

## 4. Conclusions

In this study, we examined the sensitivity of gene-knockout cell lines to known DNA damage inducers and further developed a system to evaluate their ability to induce DNA damage in a simple manner using gene-knockout cell lines derived from Nalm-6 and HeLa cells pretreated with the DNA-PKcs inhibitor NU7026. Using these classifications and systems, we also determined the ability of five natural antioxidants to induce DNA damage. Our results suggested that, of the antioxidants analyzed, sulforaphane induces SSBs or is involved in DNA crosslinking and that the major cytotoxic effect of resveratrol may not be DNA damage induction but a more complex mechanism involving DNA damage induction. Furthermore, we observed that among the flavonoids, quercetin, kaempferol, and genistein, quercetin induces DSBs, whereas kaempferol and genistein may induce DNA damage via unknown mechanisms. Therefore, the study of the DNA-damage-inducing ability of many natural antioxidants using this evaluation system will enable the elucidation of the mechanisms of the antitumor effects of natural antioxidants and contribute to their application in cancer therapy with fewer side effects.

## Figures and Tables

**Table 1:** Summary of the DSB types caused by DNA damage inducers analyzed using Nalm-6 cells.

DNA Damage Inducer	Sensitivity		Type of DSB	Reference
	NHEJ-Deficient Cells	HR-Deficient Cells		
X-ray, radiomimetic agents	More sensitive	More sensitive	Two-ended DSB	[18]
Top2 inhibitor	Hypersensitive	More sensitive	Two-ended DSB	This study, [18,28]
Alkylating agent	More sensitive	More sensitive	Two-ended DSB	This study, [21]
Top1 inhibitor	More resistant	More sensitive	One-ended DSB	This study, [18]
DNA interstrand crosslinking agent	More resistant	More sensitive	One-ended DSB	This study, [29]

Abbreviations: DSB, double-strand breaks; HR, homologous recombination; NHEJ, non-homologous end-joining; Top1, DNA topoisomerase I; Top2, DNA topoisomerase II.

## Data Availability

The data that support the findings of this study are available from the corresponding author, upon reasonable request.

## Supplementary Materials

The following are available online at https://www.mdpi.com/article/10.3390/genes14020420/s1, Figure S1: Sensitivity of Nalm-6 cells pretreatment with NU7026 to genistein.
