# Supplementary material for: A Cell System-Assisted Strategy for Evaluating the Natural Antioxidant-Induced Double-Stranded DNA Break (DSB) Style
Source: Genes (Basel). 2023 Feb 6;14(2):420. doi: 10.3390/genes14020420 (PMC9957360; doi:10.3390/genes14020420)
Supplement: Supplementary file 1 [file genes-14-00420-s001.zip › genes-2185048-supplementary.pdf]

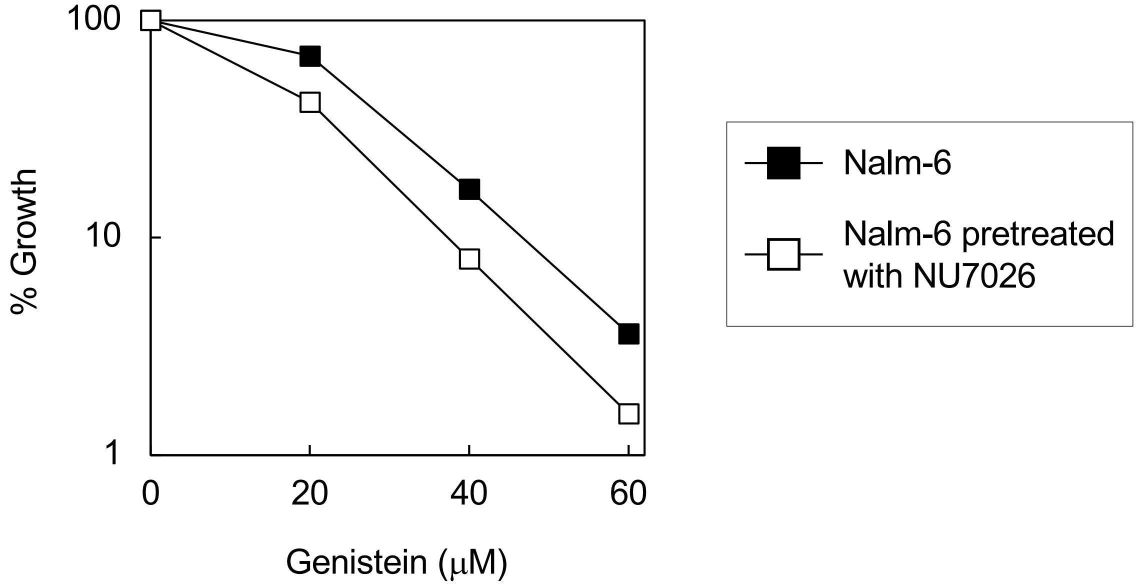

Supplementary Figure S1. Sensitivity of Nalm-6 cells pretreatment with NU7026 to genistein. Nalm-6 cells were treated with 10 μM NU7026 for 1 hour, then cultured with growth medium containing various concentration of genistein for 96 hours. Subsequently, cell growth was measured using the CellTiter-Glo Luminescent Cell Viability Assay Kit (Promega, Fitchburg, WI, USA) (n=1).
